# The Major Facilitator Superfamily Transporter HAP12 Is Critical in *Toxoplasma gondii* Survival and Virulence

**DOI:** 10.3390/ijms26083910

**Published:** 2025-04-21

**Authors:** Xiaowei Chen, Tao Tang, Huiyong Ding, Hui Dong, Shaojun Long, Xun Suo

**Affiliations:** 1 National Key Laboratory of Veterinary Public Health Security, Key Laboratory of Animal Epidemiology and Zoonosis of Ministry of Agriculture, National Animal Protozoa Laboratory, College of Veterinary Medicine, China Agricultural University, Beijing 100193, China; b20213050392@cau.edu.cn (X.C.); bs20223050479@cau.edu.cn (T.T.); dinghy@mail.sustech.edu.cn (H.D.); 2 College of Animal Science and Technology, Tarim University, Alar 843300, China; hui150614@126.com; 3 Shenzhen Key Laboratory of Pathogenic Microbes and Biosafety, and Laboratory of Zoonotic Diseases, School of Public Health (Shenzhen), Sun Yat-sen University, Shenzhen Campus, Shenzhen 518107, China

**Keywords:** *Toxoplasma gondii*, apicoplast, MFS, vaccine targets

## Abstract

As an important zoonotic pathogen, *Toxoplasma gondii* relies on a unique organelle known as the apicoplast, which has garnered significant attention as a potential drug target for anti-*Toxoplasma* therapy. To better understand the structure and function of the apicoplast, we previously constructed a membrane protein database of the apicoplast. During this process, we identified the major facilitator superfamily (MFS) transporter protein HAP12, which partially colocalizes with the apicoplast. Evolutionary analysis revealed that HAP12 is highly conserved across the *Apicomplexa* family and model organisms. HAP12 depletion impaired *T*. *gondii* invasion and survival but did not affect the stability of several key organelles, including the apicoplast. Moreover, depletion of HAP12 resulted in a characteristic delayed-death phenotype in the apicoplast. Mouse virulence assays confirmed that HAP12 is an essential protein for parasite survival. This study provides new insights into potential drug and vaccine targets for combating *Toxoplasma* infections.

## 1. Introduction

*Toxoplasma gondii*, a widely disseminated zoonotic pathogen [1], can infect various warm-blooded animals [2] and cause significant harm to humans, including brain infections [3], miscarriages in pregnant women, and fetal abnormalities [4]. The apicoplast, a unique organelle in *T*. *gondii* that is absent in host cells [5], plays a critical role in numerous metabolic activities essential for parasite stability. It has been extensively studied as a potential target for anti-*Toxoplasma* therapy [6]. The apicoplast consists of a four-layer membrane structure that originates from secondary endosymbiosis [7], which involves the engulfment of a primary plastid by an ancient apicomplexan ancestor. This endosymbiotic origin allows the apicoplast to function as a semi-autonomous organelle, performing a variety of metabolic activities within the parasite.

The apicoplast is involved in essential metabolic processes, such as de novo synthesis of fatty acids via type II fatty acid synthesis (FAS II) and the methylerythritol phosphate (MEP) pathway [8,9]. It is also involved in heme metabolism in conjunction with the mitochondrion [10] and the import of nuclear-encoded apicoplast proteins for maintaining stability [11,12]. These processes require complex molecular transport across the apicoplast’s four membranes, which is facilitated by membrane transport proteins [13,14,15]. Several membrane proteins localized to the apicoplast have been identified in *T. gondii*, including the TOC and TIC proteins responsible for transporting NEAT proteins (homologous to those in chloroplasts) [16,17]. Additionally, the apicoplast phosphate translocator (APT1) is responsible for transporting glycogen [15], the ion channel protein two-pore channels (TPC) [13], and the recently identified apicoplast pyruvate carrier (APC) [14].

These membrane proteins are crucial for both apicoplast and parasite survival, though their precise transport mechanisms across the apicoplast membranes remain unclear [6].

To better understand the apicoplast membrane structure and its associated proteins, we constructed a membrane protein database for the apicoplast and performed comprehensive identification and analysis [18]. In addition to identifying apicoplast-localized membrane proteins, we also found some non-apicoplast proteins partially colocalized with apicoplast membrane proteins [18]. Further localization studies and CRISPR-based selection led to the identification of the MFS protein HAP12 (TGGT1_226020), which partially colocalizes with the apicoplast marker ACP. Evolutionary analyses showed that HAP12 is highly conserved across the *Apicomplexa* family and model organisms. To further investigate the function of HAP12 in *T*. *gondii*, we constructed a conditional knockdown strain of HAP12-AID. Depletion of HAP12 impaired the survival and invasion of *T*. *gondii* but did not influence the stability of biotin and lipid droplets, the apicoplast, or other key organelles essential for the survival of *T*. *gondii*. Additionally, depletion of HAP12 resulted in the classical delayed-death phenotype, which only happens when the apicoplast is defective. Mice virulence assays confirmed that HAP12 is an essential protein for parasite survival, highlighting its potential as a therapeutic drug target for anti-*Toxoplasma* therapy. The identification and analysis of the HAP12 protein in this study will provide valuable insights into the interaction network of apicoplast membrane transporters and their roles in parasite metabolism.

## 2. Results

### 2.1. HAP12 Is a Highly Conserved Apicoplast MSF Protein in Toxoplasma gondii

**Figure 1:**
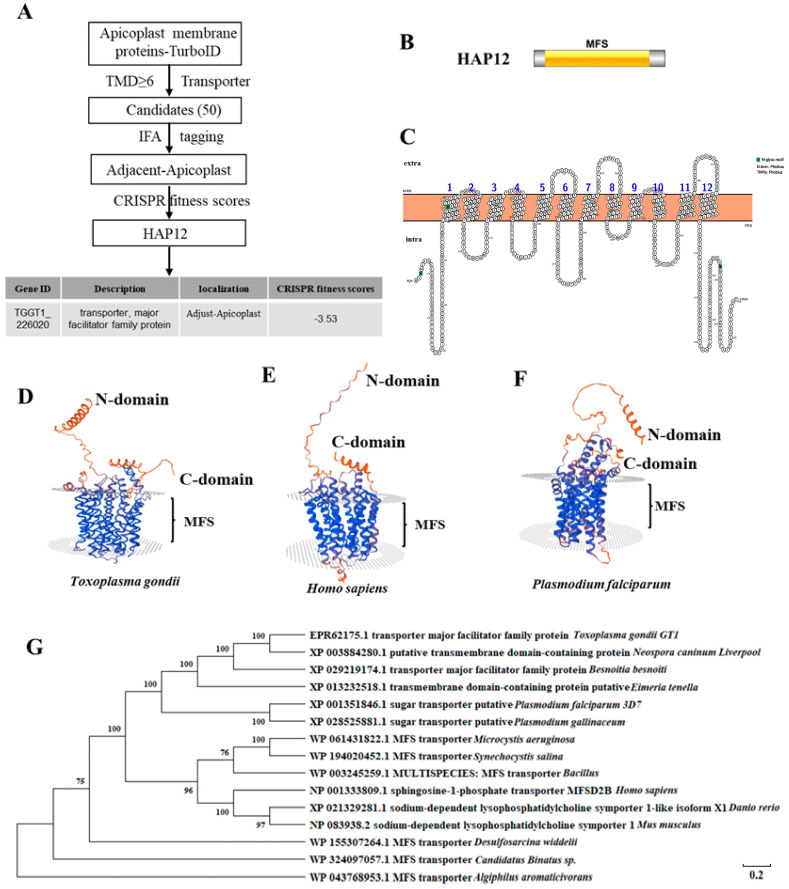
Selection and bioinformatics analysis of HAP12. (**A**) Selection of HAP12 based on the construction of the apicoplast membrane protein database. Fifty candidate proteins were selected based on the criteria of TMD ≥ 6 and transporter classification during the construction of the apicoplast membrane protein database. Colocalization with the apicoplast marker ACP was verified by IFA, and proteins adjacent to the apicoplast were identified [18]. The final candidate protein, HAP12, was selected based on CRISPR scoring. Basic information for HAP12 is provided in the table. (**B**) Domain identification of HAP12. The protein domain of HAP12 was analyzed using Uniprot (https://ibs.renlab.org/#/server/visualize?db=Uniprot, accessed on 13 January 2023) and IBS. HAP12 belongs to the MFS (Major Facilitator Superfamily), a family of transport proteins that facilitate the transport of small solutes across membranes in response to ion gradients. (**C**) Prediction and Analysis of HAP12’s Transmembrane Domains. The transmembrane structure of HAP12 was predicted using Protter (https://wlab.ethz.ch/protter/start/, accessed on 13 January 2023), which revealed transmembrane domains. (**D**–**F**) HAP12’s protein structure was predicted using SWISS-MODEL (https://swissmodel.expasy.org/, accessed on 13 January 2023), with the N-domain and C-domain representing the sequence termini and the MFS domain identified. (**D**) Predicted structure of *Toxoplasma gondii*. (**E**). Predicted structure of HAP12 in humans. (**F**) Predicted structure of HAP12 in *Plasmodium falciparum*. (**G**) Phylogenetic tree analysis. A phylogenetic tree of HAP12 was constructed and analyzed with NCBI (National Center for Biotechnology Information) and Mega X software (version 10.0.5), including members of the *Apicomplexa* family and model organisms.

Using the TurboID biotin proximity labeling method, our laboratory successfully identified apicoplast membrane proteins and constructed a relevant database [14,18]. Among the 50 candidate proteins in the database, 35 were partially colocalized with the apicoplast marker ACP [18]. Further CRISPR-based screening identified the MFS protein HAP12 (TGGT1_226020), which partially colocalized with ACP, with a CRISPR score of −3.53 (Figure 1A). According to the InterPro database search, HAP12 contains a conserved MFS domain spanning amino acid residues 61–506 (Figure 1B). Using the online tool Protter for transmembrane domain prediction, we identified 12 transmembrane domains within HAP12 (Figure 1C). A three-dimensional structural model of HAP12 revealed that both the N- and C-termini are positioned on the same side. It also confirmed the presence of a characteristic MFS transmembrane domain (Figure 1D). The three-dimensional structural model of homologous sequences from the model organisms *Homo sapiens* and *Plasmodium falciparum* (a member of the *Apicomplexa* family) revealed high structural similarity to *T*. *gondii* HAP12 (Figure 1E,F). The evolutionary analysis demonstrated that HAP12 is highly conserved across Apicomplexan species, including *Plasmodium falciparum* (GeneID:813105), *Eimeria tenella* (GeneID:25251183)*, Neospora caninum liverpool* (GeneID:13442180), and *Besnoitia besnoiti* (GeneID:40310980), as well as in model organisms such as *Homo sapiens* (GeneID:388931), *Danio rerio* (GeneID:570014) and *Mus musculus* (GeneID:76574) (Figure 1G). Multiple sequence alignments further confirmed the high sequence conservation between HAP12 and its homologs in the *Apicomplexa* family and model organisms (Figure S1).

### 2.2. Inducible Degradation of HAP12 Impairs Parasite Invasion and Survival

**Figure 2:**
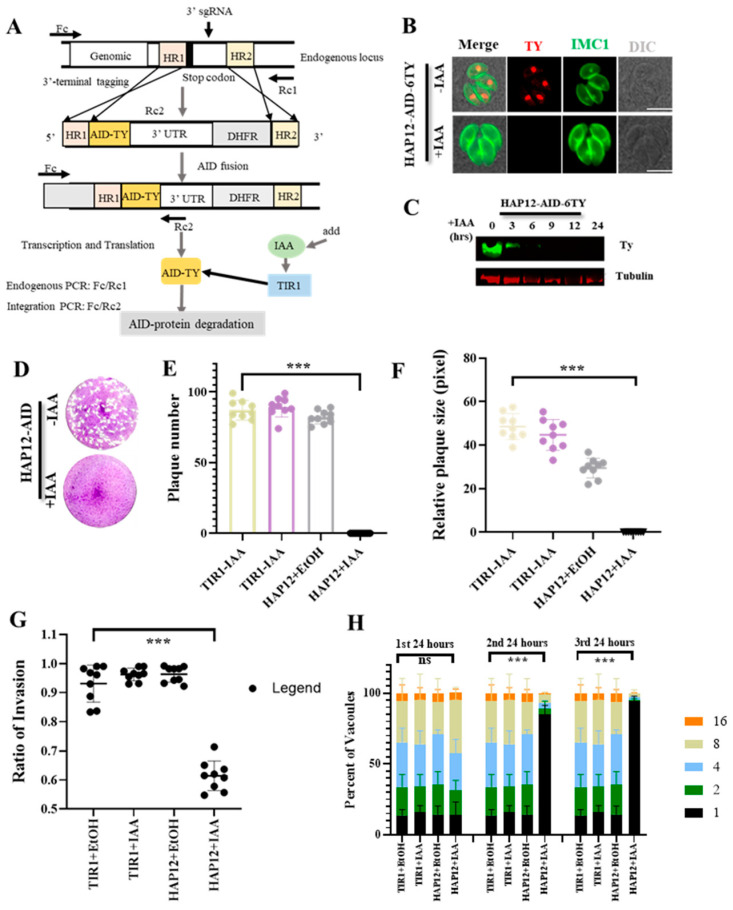
HAP12 depletion impairs phenotype of parasite. (**A**) Illustration of the endogenous tagging strategy used to fuse the AID-6TY tag to the C-terminus of the apicoplast membrane proteins in the parental TIR1 line. (**B**) IFA detection of protein depletion in HAP12-AID lines. The AID line was grown in IAA for 24 h. The lines were analyzed by IFA using anti-Ty (red) and anti-IMC1 (green) antibodies. Scale = 5 μm. (**C**) Western blot detection of protein depletion in the HAP12-AID line. The lines were induced by IAA for the indicated time periods, respectively. Actin or Tubulin served as loading controls in the assay. (**D**) Plaque formation assay for HAP12-AID lines on HFF monolayers. The lines were grown in ±IAA over 7 days, respectively. The parental lines were assayed in parallel to serve as controls for the assay. (**E**) The number of plaques formed by the AID lines was measured by ImageJ (version: Java 1.8.0_345 64-bit). (**F**) The plaque area formed by the AID lines was measured by ImageJ. Scale = 0.5 cm. Two to three independent experiments were performed in triplicate. Data are presented as mean ± standard error of the mean (SEM). (**G**) The invasion rates of HAP12-AID and TIR1 lines after treatment with or without IAA. The number of parasites per group was greater than 100, and the experiment was performed in triplicate. (**H**) Parasite replication in three rounds of culturing showed delayed death of the parasites depleted by HAP12-AID parasite lines, which were grown in IAA for 24 h, followed by scraping, harvesting, and infection for the 2nd and 3rd rounds of parasitic growth on cell monolayers in ±IAA. The samples were processed by IFA using antibodies against GAP45 prior to quantification of parasite numbers in vacuoles (at least 200 vacuoles per replicate). The data were analyzed by two-way ANOVA with Tukey’s multiple comparisons. The AID lines were compared with TIR1 in each round of experiments (1st, 2nd, and 3rd), *p* < 0.0001 (indicated by ***) was obtained for the HAP12-AID line in the 2nd and 3rd rounds. Three independent experiments were performed in triplicate.

To investigate the role of HAP12 in *T*. *gondii*, we utilized the plant auxin-inducible degron system (AID) in the RH*Δku80ΔhxgprtTIR1* background strain [19,20], tagging the C-terminal of HAP12 with an AID-TY tag (Figure 2A). After treatment with IAA for 24 h, immunofluorescence analysis showed complete loss of the TY-tag fluorescence in the HAP12-AID strain compared to the untreated control group, indicating successful degradation of HAP12 (Figure 2B). Western blot analysis also confirmed the complete degradation of HAP12 after 6 h of IAA treatment (Figure 2C), demonstrating that the HAP12-AID strain was successfully labeled and degraded upon IAA treatment. To assess the impact of HAP12 depletion on parasite survival, we cultured the HAP12-AID strain in HFF cells with and without IAA treatment for seven days. The IAA-treated HAP12-AID strain could not form visible plaques on the cell surface compared to the control group (Figure 2D). Quantification of plaque number and area revealed significant differences between the IAA-treated group and the control group (Figure 2E,F) (*p* < 0.05). These results suggest that HAP12 depletion impairs parasite survival. To investigate the effect of HAP12 depletion on parasite invasion, we measured the invasion rate of the IAA-treated HAP12-AID strain. The results showed a significant reduction in invasion efficiency compared to the untreated control (Figure 2G), indicating that HAP12 is essential for parasite invasion.

### 2.3. Inducible Degradation of HAP12 Results in a Characteristic Delayed-Death Phenotype

To further examine whether HAP12 depletion affects parasite proliferation, we counted the number of tachyzoites in parasitophorous vacuoles after 24 h of IAA treatment. No significant difference was observed in the first lytic cycle. However, in the second and third cycles, the number of tachyzoites in IAA-treated HAP12-AID vacuoles took a steep decline compared to the control groups (Figure 2H) (*p* < 0.05), indicating that HAP12 depletion impairs parasite proliferation. AID-mediated depletion of HAP12 reveals its critical role in *Toxoplasma gondii* proliferation across successive lytic cycles.

### 2.4. Inducible Degradation of HAP12 Shows No Detectable Effect on Apicoplast Integrity or Other Organelles

**Figure 3:**
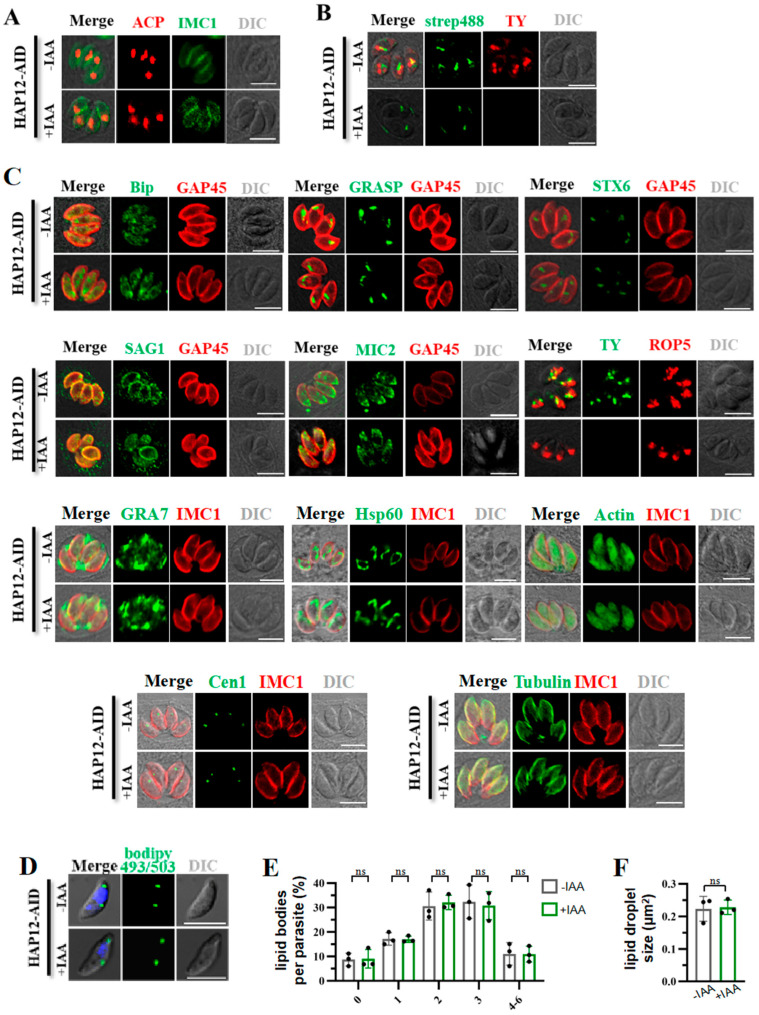
HAP12 depletion does not compromise apicoplast integrity or other organelles. HAP12-AIDs were treated with IAA for 24 h, followed by fixation of intracellular parasites. IFA was performed with antibodies. (**A**) The depletion of HAP12 had no significant effect on ACP, with no noticeable change in fluorescence intensity. Red fluorescence: ACP, Green fluorescence: IMC1. (**B**) Effect of IAA treatment on biotin uptake: the fluorescence intensity of Strep488 in the HAP12-AID line showed no significant change. Green fluorescence: Strep488, Red fluorescence: TY. (**C**) Changes in fluorescence intensity of organelle markers before and after IAA treatment. Endoplasmic reticulum marker: Bip, golgi apparatus markers (cis- and trans-Golgi): GRASP and STX6, parasite plasma membrane marker: SAG1, microneme marker: MIC2, rhoptry marker: ROP5, dense granule marker: GRA7, mitochondrion marker: Hsp60, the actin cytoskeleton marker: actin, the centriole marker: Cen1, and microtubules marker: Tubulin. Scale = 5 μm. (**D**) Changes in lipid droplets before and after IAA treatment. Lipid droplets (LDs) were stained with the neutral lipid dye BODIPY 493/503 and visualized by confocal microscopy(D). Scale = 5 μm. (**E**,**F**) The number of LDs per parasite was quantified (*n* > 200) (**E**), and the area of more than 50 LDs was measured using NIS-Elements AR software (version 4.20.00, (Build 972); LO, 32-bit) (**F**). Three independent experiments were performed with duplicate measurements in each. Data are presented as mean ± SD and were analyzed by two-way ANOVA with Tukey’s multiple comparisons test. ns, not significant.

To determine whether HAP12 depletion affects the stability of the apicoplast, we performed indirect immunofluorescence analysis (IFA) to examine the aggregation of ACP fluorescent signals in IAA-treated HAP12-AID strains. The results showed no significant change in ACP fluorescence aggregation between the IAA-treated and control groups (Figure 3A), suggesting that HAP12 depletion does not impact the stability of the apicoplast. To determine whether HAP12 depletion affects biotin uptake, we analyzed the degree of Strep488 aggregation in IAA-treated HAP12-AID parasites. No significant difference in fluorescence intensity was observed between the IAA-treated and control groups (Figure 3B), indicating that HAP12 depletion does not affect biotin uptake. We further examined the stability of other organelles by analyzing the aggregation of various organelle markers. No significant changes were observed in the degree of aggregation of the endoplasmic reticulum marker BIP, the Golgi marker GRASP and STX6, surface membrane marker SAG1, the Microneme marker MIC2, dense granule marker GRA7, mitochondrion marker Hsp60, the actin cytoskeleton marker actin, the centriole marker Cen1, and microtubule marker Tubulin after IAA treatment. Although aggregation of the rhoptry marker ROP5 significantly decreased after IAA treatment, it did not completely disappear (Figure 3C), suggesting that HAP12 depletion has no significant impact on the stability of the endoplasmic reticulum, Golgi, Microneme, surface membrane, dense granule, mitochondrion, the actin cytoskeleton, the centriole, microtubules, or rhoptry. Additionally, lipid droplets were labeled with the neutral lipid dye BODIPY 493/503 (Figure 3D), with no significant changes observed in the number or size of the lipid droplets (Figure 3E,F).

### 2.5. Inducible Degradation of HAP12 in Mice Reveals Its Critical Role in Parasite Virulence

**Figure 4:**
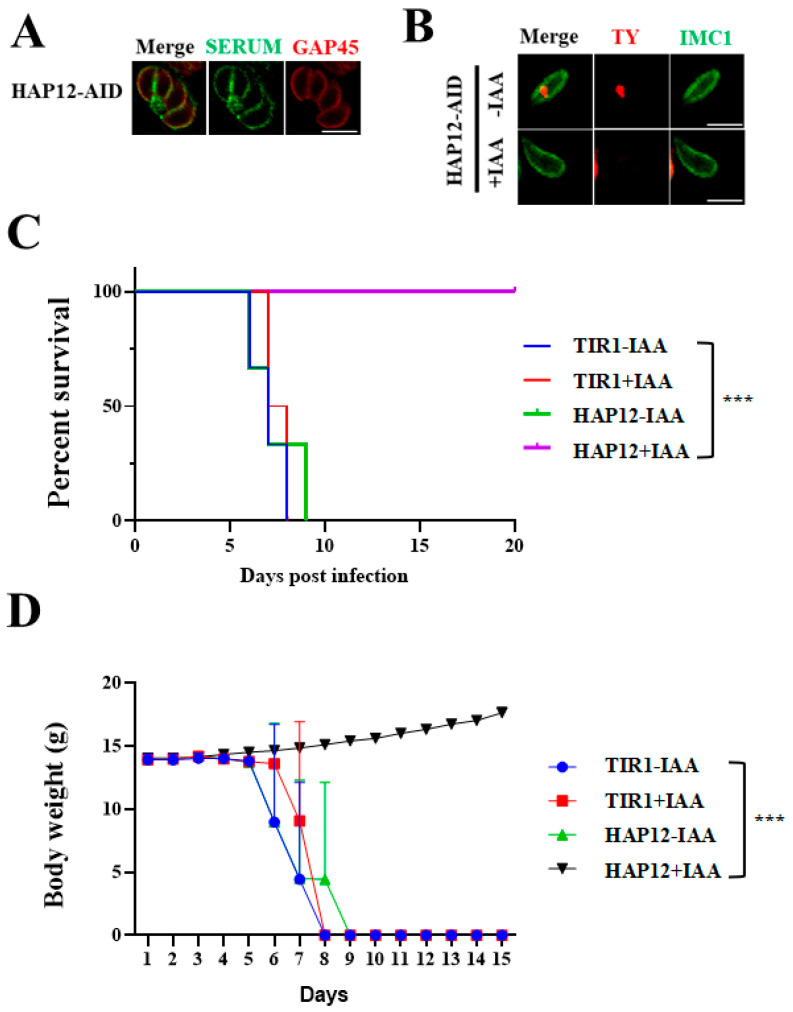
HAP12 is required for virulence in a mouse model. (**A**) Serum analysis of surviving mice revealed the presence of anti-Toxoplasma antibodies. (**B**) After infecting mice with HAP12-AID, HAP12 degradation was successfully induced by IAA treatment in vivo. Scale = 5 μm. (**C**,**D**) Virulence assays were conducted by inoculating each mouse with 100 tachyzoites. Mice in the TIR1+IAA and HAP12+IAA groups received daily intraperitoneal injections and oral administration of IAA-containing solutions. Survival rates and body weight curves of infected mice were monitored and recorded over a 20-day observation period. (***, *p* < 0.01).

To further assess the effect of HAP12 conditional knockdown on parasite growth in vivo, we infected mice with tachyzoites and analyzed their peritoneal fluid after seven days. IFA analysis showed no TY signal in the peritoneal fluid of HAP12 mice, while a strong TY signal was detected in the control group (Figure 4A), confirming successful degradation of HAP12 in vivo and validating the mouse model. Serum from the surviving mice was analyzed by IFA, and antibodies to *Toxoplasma* were detected (Figure 4B). All control mice injected with the background line TIR1 died within 10 days post-infection, while mice injected with IAA-treated HAP12-AID line survived until day 20, with a survival curve plotted (Figure 4C). The weight of mice in all groups was detected every day to ensure that the experimental data were accurate and reliable, unaffected by factors such as survival or the breeding environment (Figure 4D). These results suggest that HAP12 is an essential protein for *T*. *gondii* survival.

## 3. Discussion

The apicoplast is closely associated with other organelles such as the mitochondrion, endoplasmic reticulum, and Golgi [21], facilitating extensive metabolic interactions, material exchange, and energy transfer [22,23]. Consequently, apicoplast membrane proteins likely collaborate with other transporters to mediate substance transport. While NEAT proteins enable materials transport via vesicular fusion [24], multiple transporters are involved in this process. In model organisms, MFS family members are involved in the cation-dependent transport of substances across erythrocyte and platelet membranes in humans [25]. In zebrafish, MFS proteins facilitate transport across lipid bilayers [26]. In *Apicomplexa*, HAP12 is highly homologous to the glucose transporter of *Plasmodium falciparum* [27] and belongs to the MFS superfamily, which includes the *T*. *gondii* apicoplast membrane transporters AMT1 and AMT2 that transport pyruvate [14,18]. Given evolutionary and sequence conservation between HAP12 and other *Apicomplexa* members and model organisms, it is unlikely that HAP12 has a prokaryotic origin.

In vitro experiments showed that HAP12 depletion impairs parasite invasion and survival. Since HAP12 partially colocalizes with the apicoplast [18], we speculated that HAP12 may facilitate the transport of key metabolites, thereby influencing invasion [28]. Although the de novo fatty acid synthesis pathway in the apicoplast is considered dispensable [29], HAP12 depletion did not impact the transport of biotin, a cofactor required for key enzymes in this pathway [30]. Since tachyzoites can mediate the storage of host-derived lipids within the parasite [31], to assess whether HAP12 is involved in this process, no change in neutral lipid droplets was observed in the parasite after HAP12 depletion. This result indicates that HAP12 plays no role in the synthesis and accumulation of neutral lipids within the parasite. Furthermore, HAP12 depletion did not influence the stability of invasion-related organelles, such as the apicoplast, microneme, endoplasmic reticulum, Golgi, or rhoptry. Therefore, we hypothesized that HAP12 may regulate the transport of ions such as Ca^2+^ to influence invasion [32]. Proliferation experiments revealed a characteristic delayed-death phenotype in HAP12-depleted parasites [33], suggesting that HAP12 is involved in essential metabolic activities in the apicoplast, such as the SUF and MEP pathways [6]. This delayed-death phenotype further validates the success of TurboID biotin proximity labeling technology in identifying potential apicoplast membrane proteins [18]. However, the precise role that HAP12 plays in parasite invasion remains unclear. Given that HAP12 was identified through a membrane protein database and partially colocalizes with the apicoplast, it likely interacts with other apicoplast membrane proteins. These mechanisms require further investigation. Additionally, the transported substrates and transport mechanism of HAP12 remain to be elucidated. Although a recently identified pyruvate transporter in the apicoplast has been validated for pyruvate transport in vitro [14], the in vivo transport mechanism within the parasite remains a challenging question. A clear recognition of these unresolved aspects of HAP12 will contribute to a more comprehensive understanding of its significance in *T. gondii.*

To verify whether the MFS superfamily member HAP12 in tachyzoites shares significant immunogenic potential, similar to members of the *Cystoisospora suis* family [34], we performed mouse virulence assays. The results indicated that HAP12 is an essential protein for parasite survival. Although the precise mechanism by which HAP12 influences tachyzoites remains to be elucidated, its importance for parasite viability is unquestionable. Therefore, HAP12 has strong potential as a drug and vaccine target for the development of anti-*Toxoplasma* therapies. In *P. falciparum*, members of the MFS superfamily exhibit high levels of drug resistance [35], and play a crucial role in gamete fertilization in *P. berghei* [36]. These findings suggest that the functions of MFS transporters extend beyond substrate transport and specific parasite life cycle stages, with many roles yet to be discovered. Future research should not be limited to tachyzoites in *T. gondii*, as MFS transporters may also play important roles in bradyzoites.

## 4. Materials and Methods

### 4.1. Bioinformatics Analysis

The amino acid sequence of the HAP12 gene was obtained from the TOXODB website (https://toxodb.org/toxo/app, accessed on 13 June 2022). Online domain prediction for HAP12 was performed using InterPro (https://www.ebi.ac.uk/interpro/, accessed on 13 June 2022), and homology analysis was conducted using NCBI BLAST (BLAST: Basic Local Alignment Search Tool, https://blast.ncbi.nlm.nih.gov/Blast.cgi, accessed on 13 June 2022). Phylogenetic trees and multiple sequence alignments were constructed using MegaX and the online tool ESPript 3.0 (https://espript.ibcp.fr/ESPript/ESPript/index.php, accessed on 13 June 2022).

### 4.2. Plasmid Construction

The vector of P-CAS9-sgRNA plasmid (constructed before in our lab) was the same as before, and the sgRNA region was replaced only [37]. Several sgRNAs were designed using the online tool (http://grna.ctegd.uga.edu/), and the most efficient one was selected. The pCAS9-sgRNA plasmid was generated by *pEASY*^®^-Basic Seamless Cloning and Assembly Kit (Transgen CU201-02). The plasmid included three fragments that were amplified separately by three pairs of primers (Amp-F/Cas9-R, CAS9-F/sgRNA-F, and HAP12 sgRNA-F/Amp-R) (Table S1). The gene of interest was tagged at the terminal of 3′. The relative strategy is shown in Figure 2A. From 5′ to 3′, 18-base homology regions were chosen downstream of the sgRNA and at the end of the stop codon of the gene of interest. The template for amplification was the common-tagging plasmid PLinker-AID-6Ty-DHFR, which contains AID-6Ty-DHFR expressed downstream of the gene of interest with the primers mentioned above.

### 4.3. Parasites and Host Cell Culture

The background parasite line, RH*Δku80Δhxgprt*/*TIR1*, was previously constructed in our lab, and all gene-edited lines were derived from it [18]. All lines were cultured and propagated in human foreskin fibroblast (HFF-1) cells (ATCC, SCRC-1041) using Dulbecco’s Modified Eagle Medium (DMEM) (12800-082, Thermo Fisher Scientific, Waltham, MA, USA) supplemented with 10% inactivated fetal bovine serum, 2 mM glutamine, and 100 units of penicillin–streptomycin. Culture conditions were maintained at 37°C with 5% CO_2_, consistent with standard cell culture protocols. For phenotypic assays, the TIR1 and AID lines were cultured in HFF-1 cells with either 500 μM indole-3-acetic acid (IAA) or ethanol as a control.

### 4.4. IFA

First, HFF cells were cultured on coverslips that had been washed three times with anhydrous ethanol in a biosafety cabinet in a 24-well plate. HFF cells were then inoculated onto the coverslips. Next, endogenously labeled tachyzoites were added to wells containing confluent HFF cells and cultured for 24 h. Next, coverslips with tachyzoites were fixed with 4% paraformaldehyde (PFA) for 15 min, then the fixative was discarded. The coverslips were permeabilized with 5% BSA containing 0.25% Triton X-100 at room temperature for 15 min, then the permeabilization solution was discarded. The coverslips were blocked with 5% BSA at 37 °C for 30–45 min for antibody incubation. In a humidified chamber, coverslips were incubated with primary antibodies corresponding to endogenous labels (mouse anti-Ty 1:500, mouse anti-HA 1:500, rabbit anti-HA 1:500, rabbit anti-ACP1:500, rabbit anti-GAP451:500, rabbit anti-IMC11:500, etc.) and secondary antibodies (Alexa Fluor 594/Alexa Fluor 488 goat anti-mouse IgG, Alexa Fluor 594/Alexa Fluor 488 goat anti-rabbit IgG) at 37 °C for one hour. After incubation with primary and secondary antibodies, the coverslips were washed 3–5 times with 3% BSA containing 0.05% Tween-20. Finally, the coverslips were removed with tweezers, inverted onto a glass slide with mounting medium, and dried in the dark at 37 °C for 30 min before observation under a confocal microscope Nikon C2+ (Nikon, Tokyo, Japan).

### 4.5. Western Blot

Freshly infected T25 flasks were cultured until large parasitophorous vacuoles were observed. The parasites were then collected, resuspended in 40 μL of PBS, and mixed with 10 μL of 5× SDS loading buffer. The mixture was boiled at 100 °C for 10 min. A volume of 8–12 μL of the sample was loaded onto SDS-PAGE gel and subsequently transferred to a nitrocellulose (NC) membrane using wet transfer. The membrane was blocked with 5% skim milk at 37 °C for 1 h. After blocking, the membrane was incubated with primary antibodies (mouse anti-Ty antibody, rabbit anti-tubulin antibody) and secondary antibodies (IRDye^®^ 680RD Goat anti-Mouse IgG Secondary Antibody/IRDye^®^ 800CW Goat anti-Rabbit IgG Secondary Antibody) at 37 °C for 1 h. Following incubation, the membrane was washed 3–5 times with PBST before imaging.

### 4.6. Plaque Assay

Two hundred parasites were inoculated into a 6-well plate with confluent HFF cells. IAA or anhydrous ethanol was added to the HAP12-AID line and incubated statically in D5 medium at 37 °C for 8 days. The cells were fixed with 75% ethanol for 30 min and stained with 0.5% crystal violet for 3 h. Wells were washed with distilled water and dried at room temperature. The plaque monolayer was recorded using an HP Scanjet G4050 scanner (Hewlett-Packard, Palo Alto, CA, United States) and the number and size of plaques were measured in ImageJ (version: Java 1.8.0_345 64-bit).

### 4.7. Parasite Replication

TIR1 and the derived HAP12-AID line were cultured in a 24-well plate containing IAA for 24 h. For multiple rounds of proliferation, the lines were precultured for one or two rounds in T25 flasks with a drug supplement before being inoculated into 24-well plates. After fixing with 4% paraformaldehyde for 15 min, the samples were permeabilized with 0.25% Triton X-100 in 2.5% BSA, followed by blocking with 2.5% BSA. The samples were then incubated with rabbit anti-GAP45 primary antibody and Alexa Fluor 568 secondary antibody. Observations and counting were conducted under a Nikon C2+ microscope, with at least 100 vacuoles containing different numbers of parasites. The ratio of vacuoles containing different numbers of parasites to the total number of vacuoles examined was plotted.

### 4.8. Invasion Assay

After intracellular tachyzoites were mechanically collected, they were counted using an inverted microscope. A 0.5 mL suspension of the parasite was inoculated into a 24-well plate containing a coverslip and fresh HFF cells grown to confluence. The plate was then centrifuged at 100× *g* for 2 min at 18 °C. To facilitate parasite invasion without interference, the plate was placed in a 37 °C water bath with the bottom submerged approximately 0.5 cm in water and left undisturbed for 30 min. Following incubation, the 24-well plate was placed on ice to terminate the invasion, washed twice with cold PBS, and fixed with 4% paraformaldehyde for IFA experiments. For immunostaining, the plate was first incubated with a mouse anti-SAG1 antibody (targeting the external antigens of the parasite’s plasma membrane) (Tritonx-100 solution was not used for permeabilization). After subsequent permeabilization, the plate was incubated with the rabbit anti-GAP45 primary antibody. Coverslips were then mounted, and parasite localization was assessed using fluorescence microscopy. Parasites were visualized in three fluorescence channels: the green channel detected parasites adhering to the surface of HFF cells, the red channel displayed all parasites, and the blue channel highlighted nuclear DNA from both host cells and parasites. For each microscopic field of view, the parasites were counted in all three channels, with at least 100 parasites counted across the three channels. Therefore, the number of parasites that invaded the host cells could be obtained by subtracting the count in the green channel (Y) from the count in the red channel (X). The number of host cell nuclei was counted as Z. Finally, the number of parasites that invaded each host cell could be calculated as (X − Y)/Z.

### 4.9. In Vivo Assay

In vivo experiments were conducted on 4–6-week-old BALB/C mice to monitor the growth of *T*. *gondii*. Mice were housed under specific pathogen-free conditions and randomly divided into groups (n = 4 per group). Freshly released tachyzoites were lysed, diluted in PBS, and counted before intraperitoneal injection of 100 tachyzoites per mouse. Mice were assigned to the following groups: HAP12+IAA, HAP12-IAA, TIR1+IAA, and TIR1-IAA. The +IAA groups were fed sterile solutions containing IAA (1 mg/mL IAA, 3 mM NaOH, and 5% *w*/*v* sucrose, stirred with 2 mg/mL sugar, with a final pH of 7.4–8.0) and their stomach was injected with IAA solution (15 mg/mL IAA, 1 M NaOH, pH = 7.4) at the same time. Mice were monitored daily for appearance, weight, and responsiveness to record their health status. Blood was collected from the surviving mice and tested for Toxoplasma infection by IFA. For the deceased mice, successful induction of death was confirmed, and the experiment was repeated under the same conditions. On the fifth day, mice were euthanized, and their peritoneal fluid was extracted. Parasites were collected and adhered to poly-L-lysine-coated cell slides for IFA, using anti-IMC1/GAP45 as control proteins to observe successful degradation.

### 4.10. Statistics

Statistical analyses were performed using GraphPad Prism software (version 8.0). Data adhering to a normal distribution pattern were examined by either one-way or two-way ANOVA coupled with Tukey’s post hoc test. For smaller data sets, Student’s *t*-test was applied. A *p*-value of less than 0.05 was deemed statistically significant. The captions beneath the figures provide specific statistical details about the experiments.

### 4.11. Availability of Data and Materials

The datasets that support the conclusions of this article are incorporated into the article itself as well as the Supplementary Files.

## 5. Conclusions

In summary, this study identifies and characterizes HAP12, a MFS superfamily protein located in the apicoplast of *T*. *gondii*. Highly conserved across the *Apicomplexa* family and model organisms, HAP12 is essential for parasite survival and invasion. Its depletion results in a delayed-death phenotype when depleted. Our findings highlight the potential of HAP12 as a target for anti-*Toxoplasma* vaccines or molecular drugs. However, due to experimental limitations, the precise localization of HAP12, its transported substrates, and underlying mechanisms remain unclear. Despite these uncertainties, this study broadens our understanding of the apicoplast-associated transport network and offers new perspectives and experimental evidence for investigating material transport between the apicoplast and other organelles.

## Data Availability

Data is contained within the article and Supplementary Materials.

## Supplementary Materials

The following supporting information can be downloaded at: https://www.mdpi.com/article/10.3390/ijms26083910/s1.
