# Supplementary figures and images for: The Major Facilitator Superfamily Transporter HAP12 Is Critical in Toxoplasma gondii Survival and Virulence
Source: Int J Mol Sci. 2025 Apr 21;26(8):3910. doi: 10.3390/ijms26083910 (PMC12028005; doi:10.3390/ijms26083910)

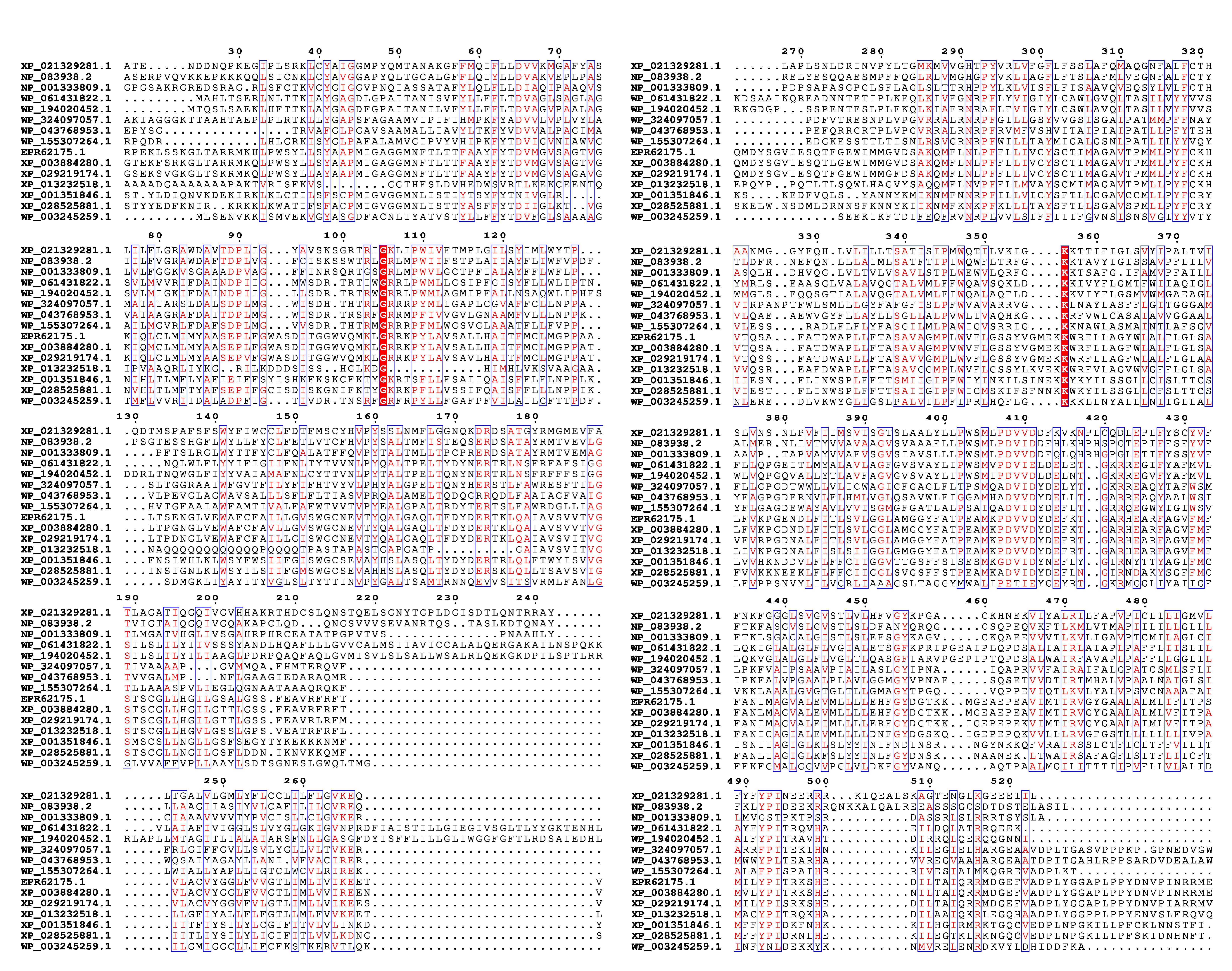

Supplement: Supplementary file 1 [file ijms-26-03910-s001.zip › Figure S.tif]
